# Nomogram to predict the outcomes of patients with microsatellite instability-high metastatic colorectal cancer receiving immune checkpoint inhibitors

**DOI:** 10.1136/jitc-2021-003370

**Published:** 2021-08-24

**Authors:** Filippo Pietrantonio, Sara Lonardi, Francesca Corti, Gabriele Infante, Maria Elena Elez, Marwan Fakih, Priya Jayachandran, Aakash Tushar Shah, Massimiliano Salati, Elisabetta Fenocchio, Lisa Salvatore, Giuseppe Curigliano, Chiara Cremolini, Margherita Ambrosini, Javier Ros, Rossana Intini, Floriana Nappo, Silvia Damian, Federica Morano, Giovanni Fucà, Michael Overman, Rosalba Miceli

**Affiliations:** 1 Department of Medical Oncology, Fondazione IRCCS Istituto Nazionale dei Tumori, Milan, Italy; 2 Medical Oncology 3, Istituto Oncologico Veneto IOV-IRCSS, Padua, Italy; 3 Unit of Clinical Epidemiology and Trial Organization, Fondazione IRCCS Istituto Nazionale dei Tumori, Milan, Italy; 4 Department of Medical Oncology, Vall d'Hebron Barcelona Hospital Campus, Vall d'Hebron Institute of Oncology (VHIO), Universitat Autonoma de Barcelona, Barcelona, Spain; 5 Department of Medical Oncology, City of Hope Comprehensive Cancer Center, Duarte, California, USA; 6 Division of Medical Oncology, Norris Comprehensive Cancer Center, Keck School of Medicine, University of Southern California, Los Angeles, California, USA; 7 Baylor College of Medicine, Houston, Texas, USA; 8 Division of Oncology, Department of Oncology and Hematology, University Hospital of Modena, PhD Clinical and Experimental Medicine (CEM), University of Modena and Reggio Emilia, Modena, Italy; 9 Multidisciplinary Outpatient Oncology Clinic, Candiolo Cancer Institute FPO-IRCCS, Candiolo, Italy; 10 Department of Medical Oncology, Fondazione Policlinico Universitario Agostino Gemelli IRCCS, Rome, Italy; 11 European Institute of Oncology (IEO), IRCCS, Milan, Italy; Department of Oncology and Hemato-oncology, University of Milan, Milan, Italy; 12 Unit of Medical Oncology 2, Azienda Ospedaliero-Universitaria Pisana; Department of Translational Research and New Technologies in Medicine and Surgery, University of Pisa, Pisa, Italy; 13 Medical Oncology 1, Istituto Oncologico Veneto IOV-IRCCS, Padua, Italy; 14 Department of Surgery, Oncology and Gastroenterology, University of Padua, Padua, Italy; 15 Department of Gastrointestinal Oncology, University of Texas MD Anderson Cancer Center, Houston, Texas, USA

**Keywords:** gastrointestinal neoplasms, immunotherapy

## Abstract

**Background:**

The efficacy of immune checkpoint inhibitors (ICIs) in patients with microsatellite instability (MSI)-high metastatic colorectal cancer (mCRC) is unprecedented. A relevant proportion of subjects achieving durable disease control may be considered potentially ‘cured’, as opposed to patients experiencing primary ICI refractoriness or short-term clinical benefit. We developed and externally validated a nomogram to estimate the progression-free survival (PFS) and the time-independent event-free probability (EFP) in patients with MSI-high mCRC receiving ICIs.

**Methods:**

The PFS and EFP were estimated using a cure model fitted on a developing set of 163 patients and validated on a set of 146 patients with MSI-high mCRC receiving anti-programmed death (ligand)1 (PD-(L)1) ± anticytotoxic T-lymphocyte antigen 4 (CTLA-4) agents. A total of 23 putative prognostic factors were chosen and then selected using a random survival forest (RSF). The model performance in estimating PFS probability was evaluated by assessing calibration (internally—developing set and externally—validating set) and quantifying the discriminative ability (Harrell C index).

**Results:**

RFS selected five variables: ICI type (anti-PD-(L)1 monotherapy vs anti-CTLA-4 combo), ECOG PS (0 vs >0), neutrophil-to-lymphocyte ratio (≤3 vs >3), platelet count, and prior treatment lines. As both in the developing and validation series most PFS events occurred within 12 months, this was chosen as cut-point for PFS prediction. The combination of the selected variables allowed estimation of the 12-month PFS (focused on patients with low chance of being cured) and the EFP (focused on patients likely to be event-free at a certain point of their follow-up). ICI type was significantly associated with disease control, as patients receiving the anti-CTLA-4-combination experienced the best outcomes. The calibration of PFS predictions was good both in the developing and validating sets. The median value of the EFP (46%) allowed segregation of two prognostic groups in both the developing (PFS HR=3.73, 95% CI 2.25 to 6.18; p<0.0001) and validating (PFS HR=1.86, 95% CI 1.07 to 3.23; p=0.0269) sets.

**Conclusions:**

A nomogram based on five easily assessable variables including ICI treatment was built to estimate the outcomes of patients with MSI-high mCRC, with the potential to assist clinicians in their clinical practice. The web-based system ‘MSI mCRC Cure’ was released.

## Introduction

The introduction of immune checkpoint inhibitors (ICIs) has produced a paradigm shift in the treatment of patients with deficient mismatch repair (dMMR)/microsatellite-instability (MSI)-high metastatic colorectal cancer (mCRC). Compared with the incremental gains achieved by several treatment options in the last 10 years, the efficacy of anti-programmed death (ligand)1 (PD-(L)1) ± anticytotoxic T-lymphocyte antigen 4 (CTLA-4) agents in this small subgroup of patients (~5%) is unprecedented. In fact, a relevant proportion of patients achieve long-term disease control, which may be the result of pharmacological tumor eradication.^1–7^

As reported in other immune-sensitive cancer types,^8–10^ a plateau of survival curves was observed in pivotal trials assessing the activity and efficacy of anti-PD-1 ± anti-CTLA-4 agents in patients with MSI-high mCRC, with reported long-term progression-free survival (PFS) rates of ~30% to 50% and~75% in patients receiving anti-PD-1 monotherapy and anti-CTLA-4 + anti-PD-1 combinations, respectively.^3–7^ In this scenario, a proportion of patients with MSI-high mCRC achieving durable responses and long-term survival outcomes may be considered potentially ‘cured’ by ICI treatment, as opposed to patients experiencing primary ICI refractoriness or short-term clinical benefit.

The development of validated, reproducible, and easy-to-use clinical tools able to discriminate between patients with MSI-high mCRC achieving long-term disease control and ICI-refractory patients represents an unmet clinical need. The early identification of potentially resistant patients with adverse clinical and biological features might be useful to timely implement more effective ICI combinations (eg, anti-CTLA-4+antiPD-1).

In the current study, we developed and externally validated a nomogram to estimate the PFS and event-free probability (EFP) in patients with MSI-high mCRC receiving ICIs and potentially assist clinicians in their clinical practice.

## Patients and methods

### Developing and validating sets

The cure model was fitted on a developing set of 163 patients with dMMR/MSI-high mCRC enrolled at six Italian academic hospitals, and the PFS prediction was validated on a set of 146 patients from five additional cancer centers. All patients were treated with anti-PD-(L)1 ± anti-CTLA-4 agents. Mismatch repair/MSI status was locally assessed through immunohistochemistry/PCR as per international guidelines.^11^

### Statistical methods

The primary study endpoint was PFS, defined as the time from the first dose of ICI treatment to disease progression or death from any cause, whichever occurred first. The observation that the PFS curves of ICI-treated patients appear to level off in the long run could support the hypothesis that patients contributing to the flat portion of the PFS curve and achieving long-term disease control may be considered potentially cured, while the others may eventually develop a progression. This was in favor of the use of a cure model,^12^ rather than the standard Cox model, to develop a nomogram for estimating the probability of being alive and progression-free at time t, that is, PFS at time t, according to a set of covariates. In detail, we used a multivariable mixture cure model where PFS at time t is defined as

PFS at time t=Probability(Be cured)+Probability(Be not cured)×Probability(Be alive and progression-free at time t if not cured), where the Probability(Be cured) was modeled through logistic regression, and the Probability(Be alive and progression-free at time t if not cured) was modeled using a Cox regression. Thus, the PFS at time t depends on the Probability(Be cured) and on the Probability(Be alive and progression-free at time t if not cured). The advantage of the cure model is that, in addition to estimation of PFS at a chosen time as in a ‘classical’ Cox model, it concomitantly allows estimation of the time-independent Probability(Be cured), both as a function of a set of prognostic factors. From hereon, the Probability(Be cured) (or ‘cure probability’, proper definition within the framework of the ‘cure models’) will be referred to as ‘EFP’. Probability(Be cured) could be more appropriate in a context where patient true status is known and ‘cure’ would mean complete pathological response or absence of disease progression with long follow-up after ICI interruption. In a prediction framework such as ours, EFP could be used more appropriately to identify patients who are likely be event-free at a certain point of their follow-up.

The nomogram to estimate PFS was developed thanks to a set of patients and externally validated by means of an independent testing set.

We chose 23 putative prognostic factors for PFS based on clinical criteria, including age, sex, primary tumor sidedness, primary tumor resection, mucinous versus non-mucinous histotype, *RAS/BRAF* mutational status, synchronous versus metachronous metastases, number of metastatic sites, presence of peritoneal metastases, lung metastases, liver metastases, bone metastases, brain metastases, lymph nodal metastases, prior adjuvant treatment, prior systemic treatment for metastatic disease, number of prior treatment lines for metastatic disease, ICI regimen (anti-PD-(L)1 monotherapy vs anti-PD-1+anti-CTLA-4 combination), ICI line, Eastern Cooperative Oncology Group Performance Status (ECOG PS) at the time of ICI treatment start, baseline lactate dehydrogenase, baseline platelet (PLT) count (×10^3^/mm^3^), and baseline neutrophil-to-lymphocyte ratio (NLR); these factors were selected for inclusion in the cure model. In the present study, we choose to select variables by resorting to the use of the random survival forest (RSF) model.^13^

As cure model results, together with the PFS nomogram picture, we report the set of scores allowing estimation of PFS at time t and the set of scores allowing estimation of the EFP based on the selected covariates. The cure model performance was evaluated by assessing calibration of PFS probabilities (internally in the developing set and externally in the validating set) and quantifying the discriminative ability by the Harrell C index (assessed as concordance between observed and predicted PFS probabilities), together with its 95% confidence interval (CI). PFS curves were estimated by the Kaplan-Meier method and statistically compared using the log-rank test. More details are provided as supplementary materials (online supplemental statistical methods).

10.1136/jitc-2021-003370.supp1 Supplementary data

## Results

The developing set included 163 patients. The validating series originally included 161 consecutive patients; 15 patients had missing information on ECOG PS (8 patients) and/or NLR (8 patients); thus, the cure model could be validated on a subset of 146 patients. Main patients and disease characteristics in the developing and validating sets are listed in table 1. A higher proportion of patients in the developing set had an ECOG PS of 0 (60.7% vs 39.7%, p<0.001), a mucinous histotype (54.0% vs 34.2%, p<0.001), lymph nodal metastases (65.6% vs 54.1%, p=0.048), ≥2 metastatic sites (59.5% vs 44.5%, p=0.005); received ≥1 prior lines of systemic treatment (77.3% vs 52.7%, p<0.001); and underwent primary tumor resection (97.5% vs 63.7%, p<0.001).

**Table 1:** Patients and disease characteristics in the developing and validating set

Characteristics	Developing set N=163 (%)	Validating set N=146 (%)	P value*
Age (years)			0.171
Median (IQR)	61 (48.5–70.5)	59 (46–69)	
Sex			0.909
Female	73 (44.8)	67 (45.9)	
Male	90 (55.2)	79 (54.1)	
Baseline ECOG PS			<0.001
0	99 (60.7)	58 (39.7)	
≥1	64 (39.3)	88 (60.3)	
Primary tumor sidedness			0.223
Left	47 (28.8)	52 (35.6)	
Right	116 (71.2)	94 (64.4)	
*RAS* and *BRAF* mutational status			0.113
All wild type	61 (37.4)	55 (40.1)	
*RAS* mutated	48 (29.4)	51 (37.2)	
*BRAF* mutated	54 (33.1)	31 (22.6)	
Primary tumor resection			<0.001
No	4 (2.5)	53 (36.3)	
Yes	159 (97.5)	93 (63.7)	
Histology mucinous			<0.001
No	75 (46)	94 (64.4)	
Yes	88 (54)	50 (34.2)	
Adjuvant treatment			0.109
No	95 (58.3)	71 (48.6)	
Yes	68 (41.7)	75 (51.4)	
NLR			1
Median (IQR)	3.2 (2.3–4.85)	3.04 (1.95–4.98)	
NLR			1
≤3	80 (49.1)	71 (48.6)	
>3	83 (50.9)	75 (51.4)	
Platelets			0.579
Median (IQR)	253 (191.5–339.5)	249 (191.8–316.5)	
Metastases presentation			0.111
Metachronous	76 (46.6)	82 (56.2)	
Synchronous	87 (53.4)	64 (43.8)	
Number of metastatic sites			0.005
0–1	66 (40.5)	81 (55.5)	
2	50 (30.7)	44 (30.1)	
≥3	47 (28.8)	21 (14.4)	
Liver metastases			0.555
No	107 (65.6)	91 (62.3)	
Yes	56 (34.4)	55 (37.7)	
Bone metastases			1
No	153 (93.9)	137 (93.8)	
Yes	10 (6.1)	9 (6.2)	
Lung metastases			0.069
No	122 (74.8)	122 (83.6)	
Yes	41 (25.2)	24 (16.4)	
Peritoneal metastases			0.194
No	97 (59.5)	98 (67.1)	
Yes	66 (40.5)	48 (32.9)	
Brain metastases			1
No	161 (98.8)	145 (99.3)	
Yes	2 (1.2)	1 (0.7)	
Lymph nodes metastases			0.048
No	56 (34.4)	67 (45.9)	
Yes	107 (65.6)	79 (54.1)	
Previous chemotherapy for metastatic disease			1
No	37 (22.7)	33 (22.6)	
Yes	126 (77.3)	113 (77.4)	
Number of prior lines for metastatic disease			<0.001
0	37 (22.7)	69 (47.3)	
1	60 (36.8)	31 (21.2)	
2	31 (19)	24 (16.4)	
≥3	35 (21.5)	22 (15.1)	
ICI line			0.454
First	37 (22.7)	42 (28.8)	
Second	61 (37.4)	48 (32.9)	
Third or more	65 (39.9)	56 (38.4)	
Regimen			0.261
Anti-CTLA-4+anti-PD-1	53 (32.5)	38 (26.0)	
Anti-PD-(L)1	110 (67.5)	108 (74.0)	

*Fisher’s exact test or Wilcoxon-Mann-Whitney test, as appropriate.

CTLA-4, cytotoxic T-lymphocyte antigen 4; ECOG PS, Eastern Cooperative Oncology Group Performance Status; NLR, neutrophil-to-lymphocyte ratio; PD-(L)1, programmed death (ligand)1.

Median follow-up, overall suvival (OS) and PFS estimates in the two sets, overall and according to regimen, are reported in online supplemental table 1; the corresponding OS and PFS Kaplan-Meier curves are shown in online supplemental figure 1. We observed that in both the development and validation series the greater proportion of PFS events occurred within 12 months (55 out of 73 overall and 48 out of 57 overall, respectively). Thus, to forecast patients’ prognosis as earlier as possible, we used 12 months as cut-point for PFS prediction. Among the 23 variables included in the RSF model, five were selected: ICI treatment type (anti-PD-1 mono vs anti-CTLA4 combo), ECOG PS at baseline (0 vs >0), NLR (modeled as binary variable ≤3 vs >3, using a literature cut-off^14^), PLT count (continuous linear term) and prior lines of treatment for metastatic disease.

10.1136/jitc-2021-003370.supp2 Supplementary data

10.1136/jitc-2021-003370.supp3 Supplementary data

**Figure 1:**
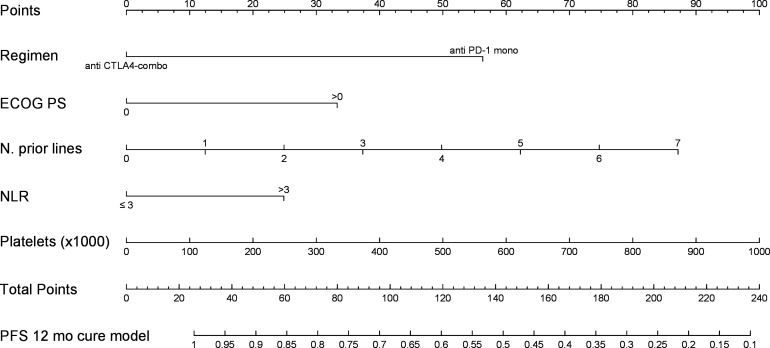
Nomogram to predict the 12-month PFS. The nomogram provides a method to calculate PFS from the date of immune checkpoint inhibitor start. To use, locate ‘regimen’ axis (anti-CTLA4-combo, anti-PD-(L)1 mono); draw a line straight up to the ‘points’ axis to determine the score associated to the regimen. Repeat for the other four variables: ECOG PS (0, >0), number of prior lines of therapy, NLR (≥3, >3) and platelet value (×1000). Sum the scores and locate the total score on the ‘total points’ axis. Draw a line straight downward to the ‘PFS 12-month cure model’ axis to obtain the 12-month PFS probability. ECOG PS, Eastern Cooperative Oncology Group Performance Status; NLR, neutrophil-to-lymphocyte ratio; PFS, progression-free survival.

**Figure 2:**
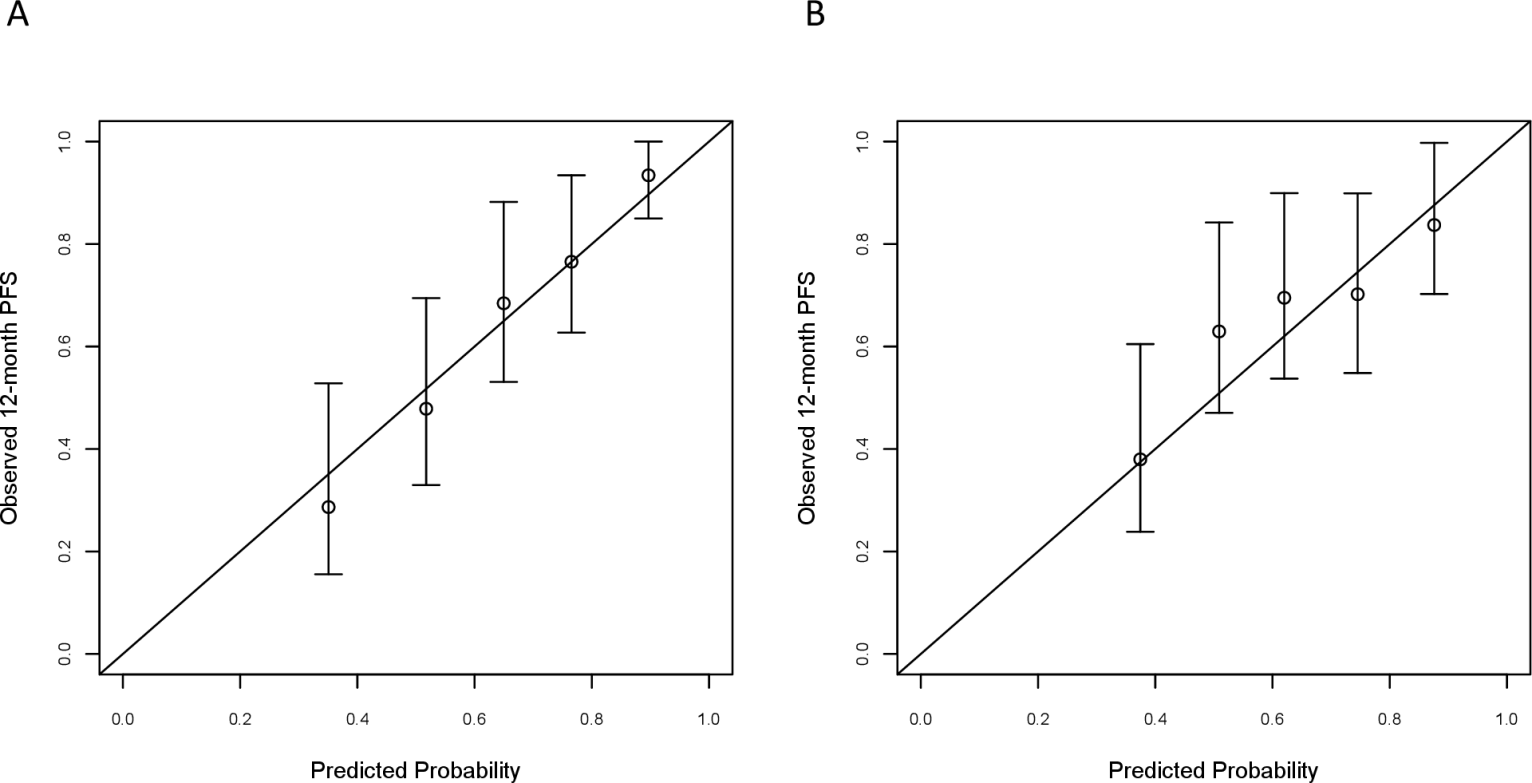
Calibration plots for internal (developing cohort, A) and external (validating cohort, B) validation of the 12-month PFS from the cure model-based nomogram. The nomogram-predicted PFS probabilities were stratified in equally sized subgroups. For each subgroup, the average predicted probability (nomogram-predicted 12-month PFS, X axis) was plotted against the observed Kaplan-Meier estimate in the subgroup (Y axis; 95% CIs of the estimates are indicated with vertical lines). Continuous line indicates the reference line, indicating where an ideal nomogram would lie. PFS, progression-free survival.

Figure 1 represents the nomogram to predict the 12-month PFS probability according to the cure model including the selected variables. The calibration of the 12-month PFS predictions was good in both the developing and validating sets (figure 2). For comparison, we show in online supplemental figure 2 the calibration plot obtained fitting a Cox model on the developing set data including the same five covariates as the cure model; Cox model predictions were not well calibrated in the group of patients with predicted 12-month PFS equal to 0.76. The Harrell C statistics were 0.707 (95% CI 0.653 to 0.762) in the developing set and 0.641 (95% CI 0.567 to 0.714) in the validating set.

10.1136/jitc-2021-003370.supp4 Supplementary data

**Figure 3:**
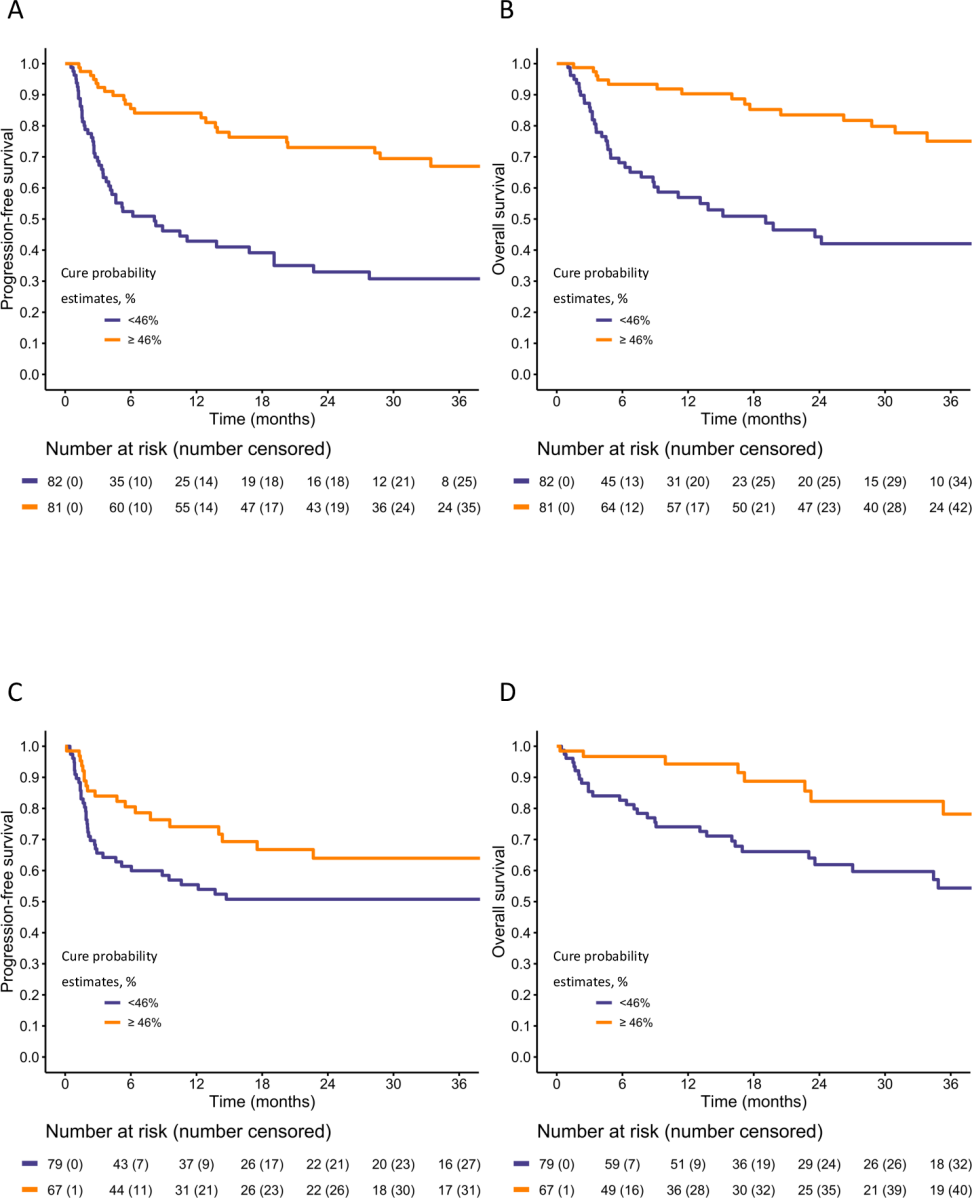
Kaplan-Meier curves for progression-free survival and overall survival in the developing set (A, C, respectively) and in the validating set (B, D, respectively). In each of the two cohorts, the patients were divided in two groups based on the median value of the cure probabilities estimated in the cure model fitted in the developing set.

Table 2 shows the two sets of scores allowing determination of the 12-month PFS and the EFP based on the nomogram covariates. The median value of the EFP (equal to 46%) allowed segregation of two well-separated prognostic groups in both developing and validating sets. In detail, in the developing set, the PFS of 12 and 36 months was 43% and 31% vs 84% and 67% in patients with EFP of <46% or≥46%, respectively (HR=3.73, 95% CI 2.25 to 6.18; p<0.0001; figure 3A). In the validating set, the PFS of 12 and 36 months was 55% and 50% vs 74% and 64% in patients with EFP of <46% or ≥46%, respectively (HR=1.86, 95% CI 1.07 to 3.23; p=0.027; figure 3B). Regarding OS, in the developing set, the OS of 12 and 36 months was 57% and 42% vs 90% and 75% in patients with EFP of <46% or≥ 46%, respectively (HR=3.86, 95% CI 2.11 to 7.06; p<0.0001; figure 3C). In the validating set, the OS of 12 and 36 months was 74% and 54% vs 94% and 78% in patients with EFP of <46% or ≥46%, respectively (HR=2.99, 95% CI 1.37 to 6.55; p=0.006; figure 3D).

**Table 2:** Score system to estimate the 12-month PFS and EFP*

Regimen	Points PFS	Points EFP	Total points PFS	PFS	Total points EFP	EFP
Anti CTLA4-combo	0	0				
Anti PD-(L)1 mono	56	24				
**Prior lines (n)**	**Points PFS**	**Points EFP**	237	0.1	134	0
0	0	0	225	0.15	127	0.05
1	12	8	213	0.2	120	0.1
2	25	17	201	0.25	114	0.15
3	37	25	190	0.3	107	0.2
4	50	34	178	0.35	100	0.25
5	62	42	166	0.4	94	0.3
6	75	50	155	0.45	87	0.35
7	87	59	143	0.5	81	0.4
**Platelets (×1000)**	**Points PFS**	**Points EFP**	131	0.55	74	0.45
0	0	0	119	0.6	67	0.5
100	10	10	108	0.65	61	0.55
200	20	20	96	0.7	54	0.6
300	30	30	84	0.75	47	0.65
400	40	40	73	0.8	41	0.7
500	50	50	61	0.85	34	0.75
600	60	60	49	0.9	28	0.8
700	70	70	37	0.95	21	0.85
800	80	80	26	1	14	0.9
900	90	90				
1000	100	100				
**ECOG PS**	**Points PFS**	**Points EFP**				
0	0	0				
>0	33	27				
**NLR**	**Points PFS**	**Points EFP**				
≤3	0	0				
>3	25	12				

*The PFS points are those shown in the figure 1 nomogram.

CTLA-4, cytotoxic T-lymphocyte antigen 4; ECOG PS, Eastern Cooperative Oncology Group Performance Status; EFP, event-free probability; NLR, neutrophil-to-lymphocyte ratio; PD-(L)1, programmed death (ligand)1; PFS, progression-free survival.

We also depicted the outcomes according to the quartiles of the EFP determined on the developing set, to investigate whether more homogeneous subgroups were still associated with differential outcomes in the context of the high efficacy of ICI in our patients’ population. Online supplemental figure 3A, C shows the PFS curves stratified according to the quartiles of the EFP in the developing set (p<0.0001) and in the validating set (p=0.024), respectively. Online supplemental figure 3B, D show the OS curves stratified according to the quartiles of the EFP in the developing set (p<0.0001) and in the validating set (p=0.004). Online supplemental table 2 shows the estimates for PFS and OS of 12 and 36 months in the subgroups identified by the median value or the quartiles of the EFP.

10.1136/jitc-2021-003370.supp5 Supplementary data

10.1136/jitc-2021-003370.supp6 Supplementary data

Online supplemental table 3 illustrates a simulation of clinical scenarios to highlight the weight of ICI treatment type on 12-month PFS and EFP estimates according to different values of cure model covariates.

10.1136/jitc-2021-003370.supp7 Supplementary data

## Discussion

In several advanced cancers, ICIs are associated with survival outcomes that are deeply different from those observed after chemotherapy or targeted therapy, with a plateau of the survival curves after a specific follow-up timepoint.^8–10^ A fraction of the patients’ population could be then considered potentially cured and, from a statistical point of view, may be subject to the same mortality of the cancer-free general population.^15^ Therefore, mixture cure models have been proposed as an alternative to traditional proportional hazard models, as the Cox model, to explore the association between survival endpoints and putative prognostic factors in a multivariable context. The advantage of cure models relies on the assumption of two different populations of patients: those who are cured and those who are ‘not cured’, thus allowing the identification of patients at high chance to be alive and event-free independently of time. Therefore, the concept of pharmacological eradication of MSI-high cancers may be better and properly investigated thanks to cure models, with relevance of this approach for trials’ design. As confirmation of the eradicating potential of immunotherapy in this molecular subgroup of patients, post-treatment surgery of metastases or early-stage primary tumors is associated with an extremely high rate of pathological complete responses, independent of the presence of radiological residuals.^16 17^ Also, patients with Response Evaluation Criteria in Solid Tumors(RECIST) very good partial responses≥50% or complete responses are usually cured, even if the best tumor response may be achieved after several months of treatment.^18^

Here we have used a multivariable mixture cure model to develop a tool for predicting the 12-month PFS probability of patients with MSI-high mCRC receiving ICIs. The timepoint for PFS prediction was chosen based on the observation that the greater proportion of PFS events occurred within 1 year, in order to allow predictions as early as possible, therefore, with specific focus on patients with low chance of being cured; this consideration is particularly true for the two groups with the worst prognosis, as appears from the PFS curves stratified according to the EFP quartiles (probability <24%, online supplemental figure 3). The EFP is focused instead on patients likely to be event-free at a certain point of their follow-up; high levels of such probability can identify those patients who will experience delayed or no progression (group with the best prognosis, probability ≥62%; online supplemental figure 3).

As regards the model covariates, we investigated the ICI treatment type and a multiplicity of variables potentially related to life expectancy and commonly used in clinical practice, and then used a random forest model leading to select five easy-to-collect variables: anti-PD-(L)1 monotherapy versus anti-CTLA-4 combination, number of prior treatment lines for metastatic disease, ECOG PS, NLR, and PLT.

ECOG PS and blood-based biomarkers are known independent prognostic variables identified by standard Cox models in available datasets of patients with MSI-high mCRC,^19 20^ and these parameters were validated also by our methodological approach. However, despite the presence of poor prognostic characteristics such poor PS and laboratory evidence of systemic inflammation, a relevant proportion of patients can still achieve long-term survival thanks to immunotherapy.^21^

ICI treatment type has a relevant impact on PFS and EFP estimates, especially in high-risk patients as identified by cure model covariates (online supplemental table 3, lines 7 and 8).

Anti-PD-(L)1 mono-immunotherapy has a manageable safety profile but is associated with a ~30% rate of primary refractoriness,^7^ whereas non-randomized trials showed that anti-CTLA-4-containing dual immunotherapy is associated with limited rates of primary resistance, at a price of higher risk of immune-related adverse events.^5 6^ The better outcomes achieved by an anti-CTLA-4-based dual regimen may be even more relevant for patients’ subgroups with poor prognosis. Therefore, a careful balance between the risk of disease progression or death and the potential occurrence of severe toxicities needs to be carried out in clinical practice. In this perspective, a nomogram encompassing both baseline variables and treatment type may be useful to inform therapeutic choices by weighing the predicted benefit of a given ICI regimen against the risk of toxicity on an individual basis. Moreover, in the perspective of future clinical trials, patients who are more likely to achieve long-term benefit from anti-PD-(L)1 monotherapies alone could be spared from unnecessary toxicity.

To date, ongoing clinical trials are aimed at investigating several novel approaches including immunotherapy combinations and immunotherapy in combination with chemotherapy.^22 23^

The clinical usefulness of our tool should be validated in ongoing randomized clinical trials investigating anti-CTLA-4-based dual immunotherapy versus single agent and could be tested in other randomized trials with combination strategies. A free app called MSI mCRC Cure has been developed as a web-based online system (freely downloadable online); it allows the user to calculate the 12-month PFS and the EFP based on the combination of the nomogram covariates in individual patients.

Our study has some clear limitations, including its retrospective nature and patients’ and treatment heterogeneity. From a statistical viewpoint, resorting to a global beforehand variable selection using the RSF model^13^ and not allowing covariate differentiation between the two cure and failure probability model components could be thought of as limitations. However, we think that this strategy is consistent with the ultimate end of the mixture cure model, that is, to estimate a survival probability. Moreover, using the same covariates in both the cure and failure probability models can be an advantage to generalize model estimates and to identify which model covariates are more associated to the cure component and less to the failure component and vice versa, which is useful from a clinical viewpoint for decision-making activities. Another potential limitation lies in that the 12-month timepoint may be early to consider a subject as cured, despite recent evidence on the maintenance of the plateau of the survival curves at the 4 year follow-up update of the ipilimumab plus nivolumab cohort of the CheckMate-142 trial.^24^ However, we selected the 12-month timepoint to allow predictions as early as possible, with a specific focus on patients with high chance of resistance to ICIs. Moreover, the cure model was subsequently fitted based on PFS independently of a specific timepoint.

In conclusion, thanks to a large multicenter collaborative effort, we developed a nomogram based on five easily assessable clinical variables to estimate 12-month PFS, giving both a scoring system to calculate the 12-month PFS and a scoring system to estimate the time-independent EFP of patients with MSI-high mCRC receiving ICIs. Prospective validation of the nomogram is required, particularly to assess its discriminative performance in selected patients’ subgroups.

## Data Availability

Data are available upon reasonable request.
