# Supplementary material for: Nomogram to predict the outcomes of patients with microsatellite instability-high metastatic colorectal cancer receiving immune checkpoint inhibitors
Source: J Immunother Cancer. 2021 Aug 24;9(8):e003370. doi: 10.1136/jitc-2021-003370 (PMC8386222; doi:10.1136/jitc-2021-003370)
Supplement: Supplementary data [file jitc-2021-003370supp001.pdf]

Supplementary Statistical methods

1. Cure model

As it was biologically plausible that some of our MSI-high mCRC patients were cured after ICI treatment, a cure model was chosen as an alternative to conventional survival analysis models, such as the Cox one (1,2). We used a multivariable mixture cure model, where PFS at time t is defined as:

$$PFS(t) = \pi + (1-\pi) * PFS_u(t) \quad (1)$$

where:

PFS(t)= PFS at time t

π = Probability of being cured ("cure probability")

$1-\pi$ = Probability of being not cured

$PFS_u(t)$= Probability of being alive and progression-free at time t if not cured.

In the present study we choose to select variables not considering the mixture nature of the underlying PFS time distribution, *i.e.* not considering that a certain proportion of patient population is 'cured'and the complementary proportion is not cured, as in the formula (1), by resorting to the use of random survival forest (RSF) model.

2. Random survival forest

Random forests (RFs) are supervised learning algorithms generating a "forest", i.e. an ensemble of decision trees grown on several bootstrap samples coming from the development set, and merging them to get more accurate and stable predictions. Random Survival Forests (RSFs) (3, 4) are a RF extension to analyze right censored time to event data. The forest of survival trees is grown using a log-rank splitting rule to select the optimal candidate variables; at each node of the tree, a given number random subset of explanatory variables is chosen, and the best split is calculated only within this subset. The chances of variable inclusion were weighted in order to to select ICI treatment at 99.9%.

RF algorithms appear very powerful in a lot of different applications to handle many predictor variables and effectively managing their collinearity, to select variables, and generate predictions. However, unlike popular survival models such as the semiparametric Cox model, RSF does not require the explicit specification of the functional form of the relationship between survival and covariates. Therefore, there are no explicit p-value/significance test for variable selection, and for this reason it is difficult to elaborate a table with the RSF model results.

The RSF model was chosen after parameters tuning to select the best performances in terms of out-of-bag (OOB) prediction error (estimated using the OOB sample, i.e. the set of observations which

are not used for building the current tree), based on of minimal depth (the lowest the best) and variable importance (estimated on OOB sample; the highest the best) criteria. In addition, it was introduced a weight system to adjust variable selection net of ICI treatment.

3. Software

All statistical analyses were performed using the R software (R Development Core Team (2007). R: A language and environment for statistical computing. R Foundation for Statistical Computing, Vienna, Austria. ISBN 3-900051-07-0, http://www.R project.org (accessed July 21, 2021))

References

1. Jia X, Sima CS, Brennan MF, Panageas KS. Cure models for the analysis of time-to-event data in cancer studies: Cure Models in Cancer Studies. J Surg Oncol. 2013;108(6):342-347. oi:10.1002/jso.23411.

2. Othus M, Barlogie B, Leblanc ML, Crowley JJ. Cure models as a useful statistical tool for analyzing survival. Clin Cancer Res 2012; 18:3731-6.

3. Ishwaran H, Kogalur UB, Blackstone EH, et. al. Random Survival Forests. Ann Appl Stat 2008; 2:841–860.

4. Ehrlinger J. ggRandomForests: Exploring random forest survival." preprint arXiv:1612.08974 (2016) https://arxiv.org/pdf/1612.08974.pdf
