# Supplementary material for: Nomogram to predict the outcomes of patients with microsatellite instability-high metastatic colorectal cancer receiving immune checkpoint inhibitors
Source: J Immunother Cancer. 2021 Aug 24;9(8):e003370. doi: 10.1136/jitc-2021-003370 (PMC8386222; doi:10.1136/jitc-2021-003370)
Supplement: Supplementary data [file jitc-2021-003370supp002.pdf]

Supplementary Table 1. Median follow-up, Overall Survival and Progression-Free Survival estimates in the developing and validating sets, in the whole series and according to treatment type.

	**Developing set**	**Validating set**	**p value at log-rank test**
Median follow-up (interquartile range), months	30.8 (11.6-44.8)	27.9 (8.4-42.8)	
Overall survival % (95% confidence interval)			
Whole series, 12-month	73.8 (66.9-81.4)	82.5 (76.1-89.5)	0.236
Whole series, 36-month	58.9 (50.6-68.6)	64.0 (54.8-74.8)	
Anti-CTLA-4 + anti-PD-1, 12-month	86.2 (72.2-96.2)	92.0 (81.8-100.0)	0.407
Anti-CTLA-4 + anti-PD-1, 36-month	73.3 (61.8-86.9)	82.3 (67.8-100.0)	
Anti-PD(L)-1, 12-month	67.0 (58.0-77.4)	79.6 (72.0-88.1)	0.094
Anti-PD(L)-1, 36-month	51.4 (40.9-64.5)	58.3 (47.6-71.5)	
Progression-free survival % (95% confidence interval)			
Whole series, 12-month	63.7 (56.4-71.9)	63.8 (56.1-72.7)	0.656
Whole series, 36-month	49.2 (41.2-58.8)	56.7 (48.4-66.3)	
Anti-CTLA-4 + anti-PD-1, 12-month	80.4 (70.2-92.1)	75.9 (62.6-92.0)	0.849
Anti-CTLA-4 + anti-PD-1, 36-month	67.4 (55.4-82.0)	71.5 (57.0-89.6)	
Anti-PD-(L)1, 12-month	55.0 (46.0-65.9)	60.2 (51.3-70.7)	0.370
Anti-PD-(L)1, 36-month	39.4 (29.9-52.0)	52.2 (42.8-63.6)	

Supplementary Table 1 Legends: **CTLA-4**: Cytotoxic T-Lymphocyte Antigen 4; **PD-(L)1**: Programmed-death (ligand)-1
