# Supplementary figures and images for: Nomogram to predict the outcomes of patients with microsatellite instability-high metastatic colorectal cancer receiving immune checkpoint inhibitors
Source: J Immunother Cancer. 2021 Aug 24;9(8):e003370. doi: 10.1136/jitc-2021-003370 (PMC8386222; doi:10.1136/jitc-2021-003370)

## Supplementary Figure 1

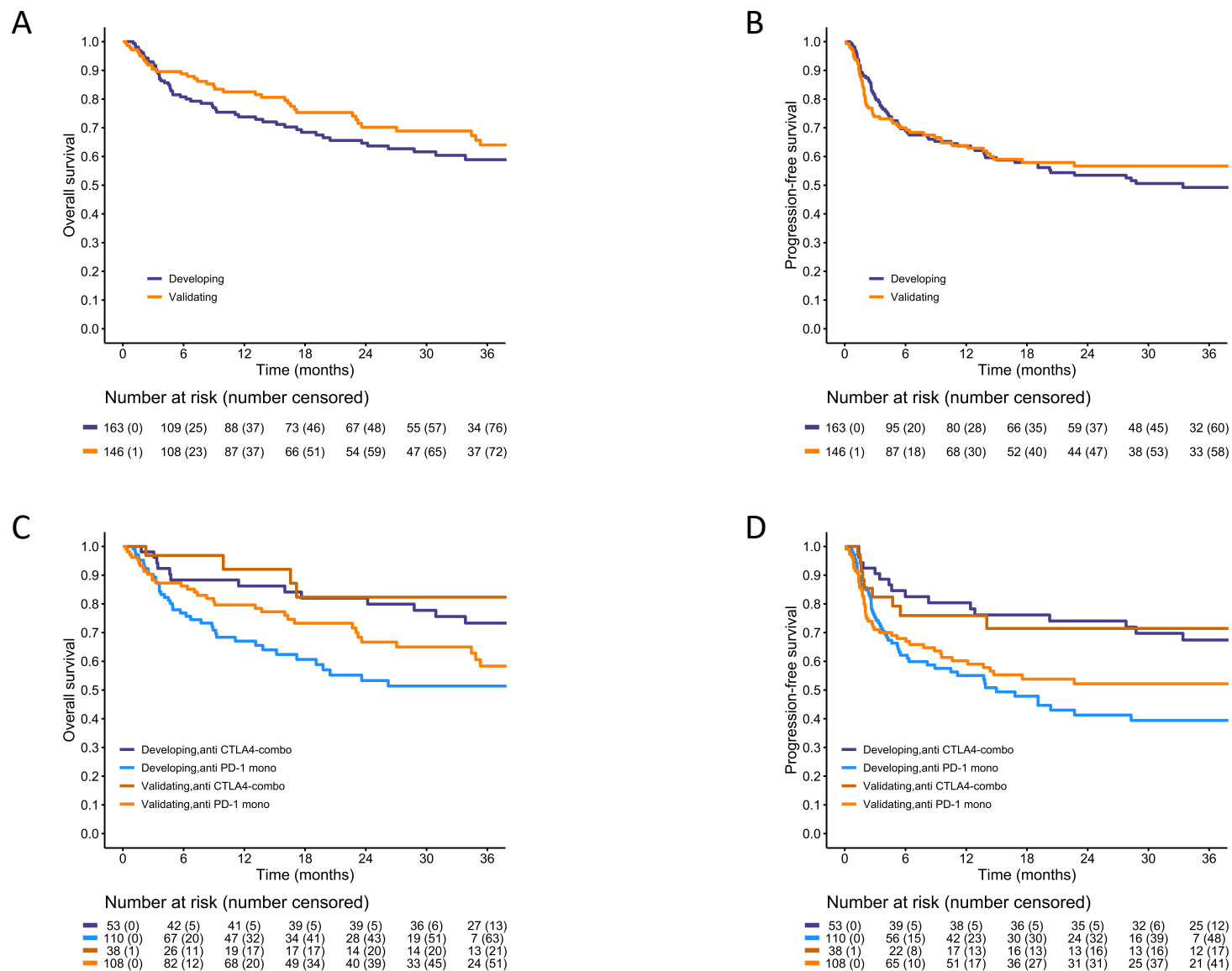

Supplement: Supplementary data [file jitc-2021-003370supp003.pdf]

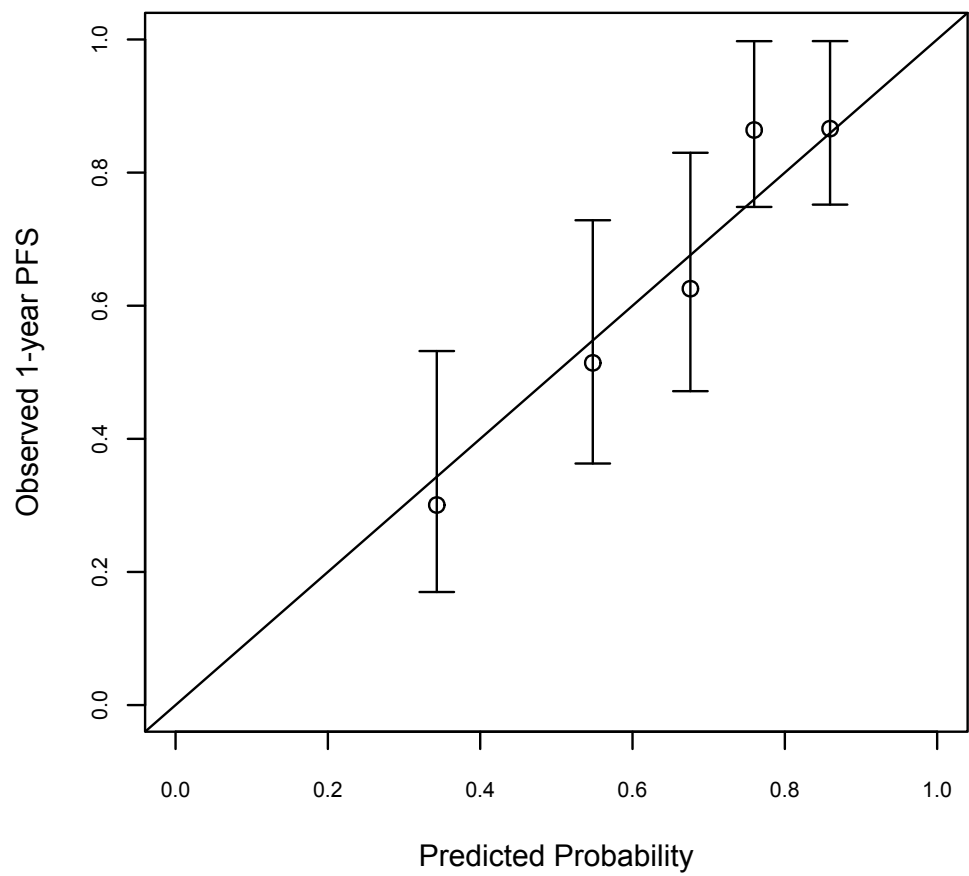

Supplement: Supplementary data [file jitc-2021-003370supp004.pdf]

Supplementary Figure 3

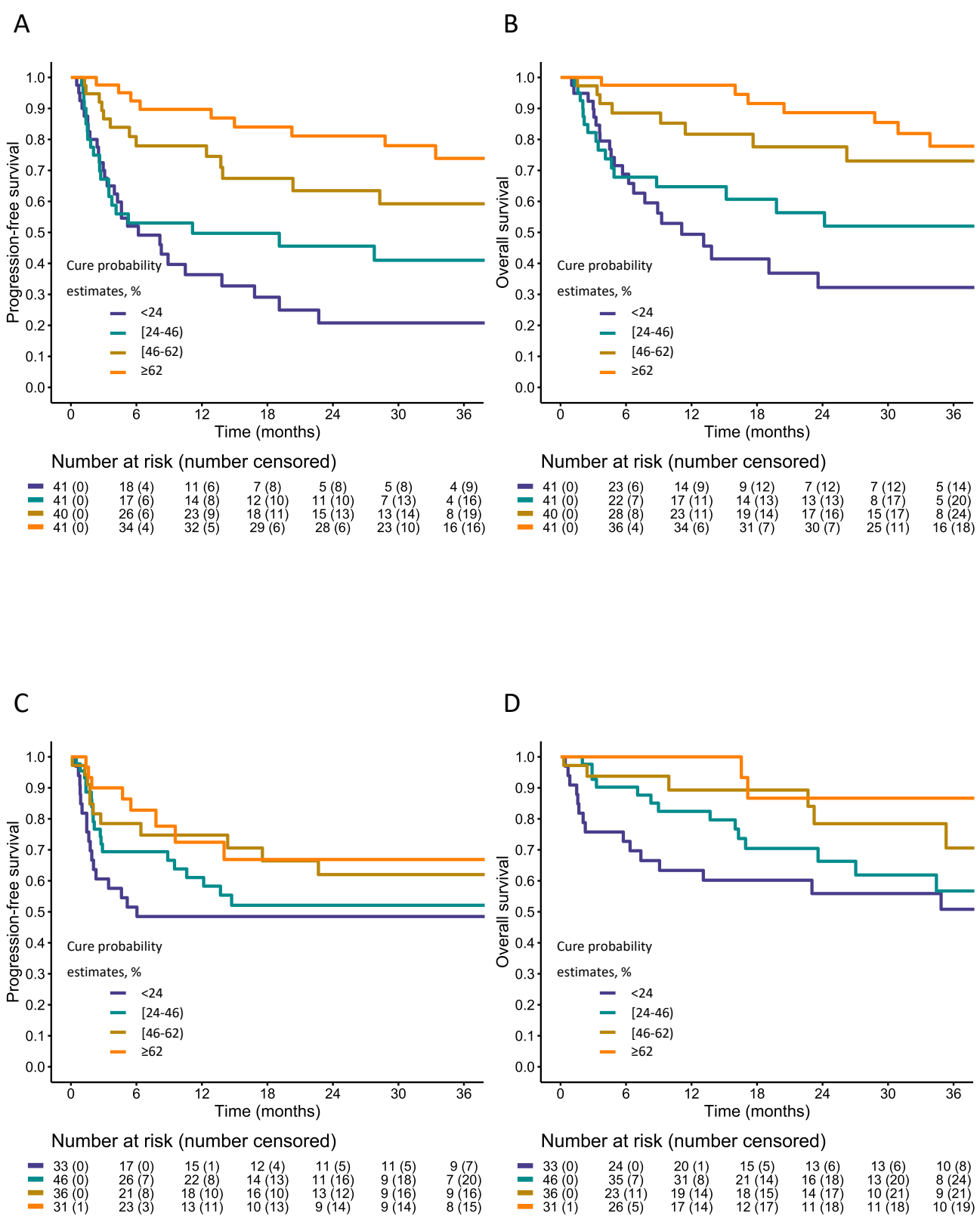

Supplement: Supplementary data [file jitc-2021-003370supp005.pdf]
