# Supplementary material for: Nomogram to predict the outcomes of patients with microsatellite instability-high metastatic colorectal cancer receiving immune checkpoint inhibitors
Source: J Immunother Cancer. 2021 Aug 24;9(8):e003370. doi: 10.1136/jitc-2021-003370 (PMC8386222; doi:10.1136/jitc-2021-003370)
Supplement: Supplementary data [file jitc-2021-003370supp006.pdf]

Supplementary Table 2. Twelve and 36-month PFS and OS according to the median value or quartiles of the event-free probability calculated on the development set.

Cure probability estimates, %	12-month PFS, % (95% CI)	36-month PFS, % (95% CI)	12-month OS, % (95% CI)	36-month OS, % (95% CI)
**Median**				
**<46**	42.9 (32.8-56.0)	30.8 (21.1-44.9)	56.9 (46.4-69.9)	42.1 (31.0-57.1)
**≥46**	84.1 (76.2-92.8)	67.0 (56.2-79.8)	90.3 (83.7-97.4)	75.0 (64.5-87.3)
**Quartiles**				
**<24**	36.4 (23.5-56.3)	20.8 (10.2-42.3)	49.4 (35.1-69.6)	32.2 (18.7-55.7)
**[24-46)**	49.7 (35.9-68.9)	41.0 (26.9-62.6)	64.7 (50.9-82.4)	52.0 (36.8-73.5)
**[46-62)**	77.9 (65.5-92.7)	59.2 (43.9-79.9)	81.7 (69.4-96.2)	73.0 (58.3-91.6)
**≥62**	89.7 (80.6-99.8)	73.9 (60.3-90.6)	97.5 (92.8-100)	77.8 (64.3-94.1)

Note: Square brackets indicate that the number is included in the interval, whereas round brackets indicate that the number is excluded from the interval.

Supplementary table 2 legends: CI: confidence interval; **OS**: overall survival; **PFS**: progression-free survival
