# Supplementary material for: Nomogram to predict the outcomes of patients with microsatellite instability-high metastatic colorectal cancer receiving immune checkpoint inhibitors
Source: J Immunother Cancer. 2021 Aug 24;9(8):e003370. doi: 10.1136/jitc-2021-003370 (PMC8386222; doi:10.1136/jitc-2021-003370)
Supplement: Supplementary data [file jitc-2021-003370supp007.pdf]

Supplementary Table 3. Twelve-month Progression-free Survival (PFS) and Event-Free Probability (EFP) in simulations of clinical scenarios according to different values of cure model covariates including the ICI treatment types.

Regimen	ECOG PS	N. prior lines	NLR	Platelets (x1000)	PFS	EFP
1. Anti-CTLA-4 + anti-PD-1	0	0	≤3	200	0.948	0.853
2. Anti-PD-(L)1	0	0	≤3	200	0.820	0.707
3. Anti-CTLA-4 + anti-PD-1	>0	1	≤3	200	0.850	0.619
4. Anti-PD-(L)1	>0	1	≤3	200	0.604	0.403
5. Anti-CTLA-4 + anti-PD-1	>0	1	≤3	400	0.772	0.406
6. Anti-PD-(L)1	>0	1	≤3	400	0.492	0.222
7. Anti-CTLA-4 + anti-PD-1	>0	5	>3	400	0.483	0.111
8. Anti-PD-(L)1	>0	5	>3	400	0.192	0.049

Supplementary Table 3 Legends: CTLA-4: Cytotoxic T-Lymphocyte Antigen 4; **ECOG PS**: Eastern Cooperative Oncology Group Performance Status; **N**: number; **NLR**: neutrophil-to-lymphocyte ratio; **PD-(L)1**: Programmed-death (ligand)-1
